# The association of chronic apical periodontitis and endodontic therapy with atherosclerosis

**DOI:** 10.1007/s00784-013-1156-3

**Published:** 2013-12-12

**Authors:** Johannes Petersen, Eva-Maria Glaßl, Parinaz Nasseri, Adriano Crismani, Anna K. Luger, Elisabeth Schoenherr, Kristina Bertl, Bernhard Glodny

**Affiliations:** 1 Department of Radiology, Innsbruck Medical University, Anichstrasse 35, 6020 Innsbruck, Austria; 2 Department of Orthodontics, Innsbruck Medical University, Innsbruck, Austria; 3 Department of Oral and Maxillofacial Surgery, Paracelsus Medical University, Salzburg, 5020 Austria; 4 Bernhard Gottlieb School of Dentistry, Medical University of Vienna, Vienna, Austria

**Keywords:** Atherosclerosis, Periapical periodontitis, Marginal periodontitis, Multidetector computed tomography

## Abstract

**Objectives:**

Chronic apical periodontitis (CAP) appears to be a risk factor for coronary heart disease. The aims of the study were to estimate the significance of AP for the atherosclerotic burden and to examine the potential effect of endodontic treatment.

**Materials and methods:**

The whole-body computed tomography (CT) examinations of 531 patients with a mean age of 50 ± 15.7 years were evaluated retrospectively. The atherosclerotic burden of the abdominal aorta was quantified using a calcium scoring method. The parameters of periodontitis were measured using the CT scan.

**Results:**

The patients had a total of 11,191 teeth. The volume of the aortic atherosclerotic burden for patients with at least one CAP lesion was 0.32 ± 0.92 ml, higher than for patients with no CAP (0.17 ± 0.51 ml; *p* < 0.05). The atherosclerotic burden increased with age and number of CAP lesions without root canal treatment, but not with number of CAP lesions with endodontic treatments (*p* < 0.05 each). In logistic regression models, age (Wald 90.8), CAP without endodontic treatment (Wald 39.9), male gender (Wald 9.8), and caries per tooth (Wald 9.0) correlated positively and the number of fillings (Wald 11) correlated negatively with the atherosclerotic burden (*p* < 0.05 each). Apical radiolucencies in teeth with endodontic treatment were irrelevant with respect to atherosclerosis.

**Conclusions:**

CAP correlated positively with the aortic atherosclerotic burden. In regression models, CAP without endodontic treatment was found to be an important factor, not however apical radiolucencies in teeth with endodontic treatment.

**Clinical relevance:**

Further research is needed to clarify the possible clinical significance of these associations.

## Introduction

Just a few years after the first indications that inflammatory diseases and infections might be associated with the occurrence of cardiovascular events such as myocardial infarction [1–3], there is increasing evidence that they might even be the cause of such events [4, 5]. As a chronic oral disease, marginal periodontitis was also considered to be a potential risk factor for acute myocardial infarction [6, 7]. This hypothesis gained support in the Atherosclerosis Risk in Communities study, in which a correlation was found between the extent of marginal periodontitis and the intima–media thickness of the carotid artery measured using ultrasound [8]. The significance of marginal periodontitis as an independent risk factor for the progression of the intima–media thickness of the carotid artery has also been demonstrated [9, 10]. The level of evidence for a causal relationship between marginal periodontitis and atherosclerosis is high, as some of the studies were prospective [8, 9]. The available data on chronic apical periodontitis (CAP) [11], primarily caused by pulpal infection [12–14], is much less reliable. In one study, the period before the occurrence of coronary heart disease was found to be shorter for persons under age 40 with CAP, but not for those who were older [15]. In two other studies with similar evidence, endodontic treatment was used as a surrogate parameter for CAP [16, 17], and no correlation was found in a fourth study [18]. There is evidence from two recent studies that “lesions of endodontic origin” may be associated with coronary heart disease [19] and that the number and extent of carious lesions may be associated with atherosclerosis [20]. The aim of endodontic treatment is to reduce and heal pulpal infection. Ideally, this can halt or even reverse processes of chronic inflammation manifested as CAP. While it can be expected that CAP correlates positively with the atherosclerotic burden, it may be that the association is attenuated or even reversed by endodontic treatment, since endodontic treatment interrupts the chain of infection and inflammation. The objective of this study was therefore to estimate for the first time the extent of the association of CAP and endodontic treatment with atherosclerosis in a large patient population using an objective calcium scoring method to quantify the atherosclerotic burden [21].

## Materials and methods

The retrospective cross-sectional study was conducted after being approved by the institutional ethical review board of Innsbruck Medical University. The guidelines of the World Medical Association from the Declaration of Helsinki were complied with. A total of 531 patients, mean age 50 ± 15.7 years (range 8–89 years; 259 females/272 males), who had had a whole-body computed tomography (CT) scan were included in the study. These scans used protocols designed to image osseous structures to assess arthritis (327 patients, 61.6 %), identify tumors in suspected neoplastic disease (87 patients, 16.4 %), stage tumors (60 patients, 11.3 %), or evaluate trauma (57 patients, 10.7 %).

The 0.625-mm collimated source images were available for all examinations. The examinations were conducted on a 16- or 64-slice spiral CT scanner (General Electric LightSpeed or VCT, Milwaukee, WI, USA). The two scanners were calibrated daily using phantoms to ensure constancy of the equipment.

The jaws were imaged in display fields of view with diameters between 12 and 25 cm with a matrix of 512 × 512 pixels each. The resulting resolution was between 0.23 and 0.48 mm in the *x* and *y*-axes. The slice interval was 0.2 mm. As a minimum, coronal and sagittal reconstructions with a slice width of 0.5 mm and a slice interval of 0.2 to 0.6 mm were available. In addition, several curved 3D reconstructions were made perpendicular to the roots of the teeth in order to simulate panoramic radiographies. An Advantage Windows workstation, version 4.4 (General Electric AW4.4, Milwaukee, WI, USA) was used.

**Fig. 1:**
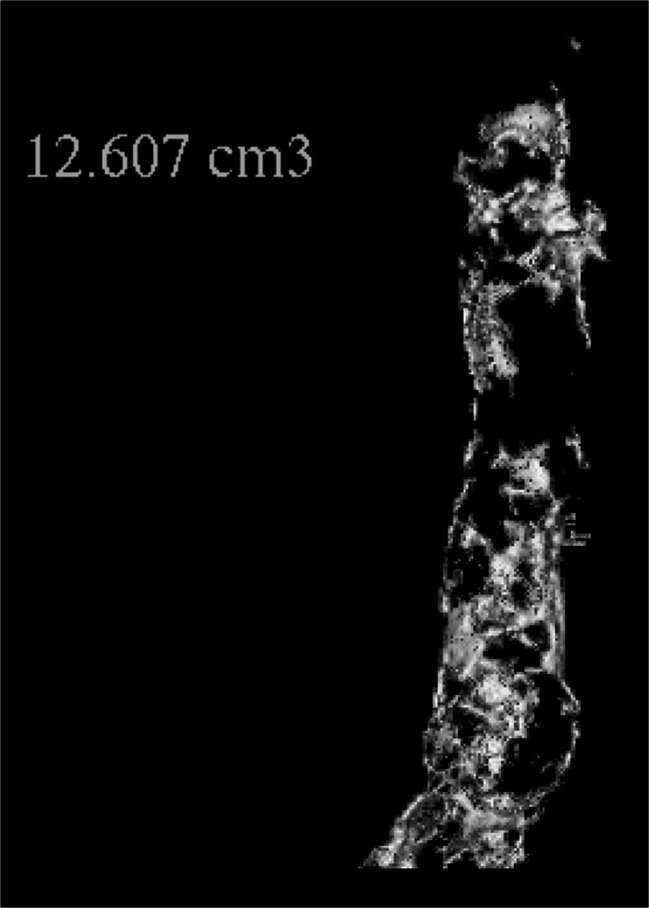
Illustration of a 3D volume rendering reconstruction of the abdominal aorta after elimination of soft tissue, blood, and bones. Only calcified atherosclerotic plaques with a density higher than 600 HU remain

The atherosclerotic burden was quantified at a defined segment of the abdominal aorta between the origin of the celiac trunk and the bifurcation using a method also suitable for volume data sets acquired by helical scanning from CTs not designed for calcium scoring [21]. To do this, a lower density threshold of 160 HU was introduced at the Advantage Windows 3D workstation in a volume rendering reconstruction to eliminate soft tissue overlay. Then the denser structures such as bones or any clips were cut out using a scissors tool so that only the aorta remained. After all structures with a density of less than 600 HU (Fig. 1) were excluded electronically, the volume of the remaining calcified plaques was automatically measured [22]. This means of measuring calcium plaques is an accepted method for a general assessment of cardiovascular status. In a final step, the measurements were calibrated to the patient’s body size, as the length of the abdominal aorta between the origin of the celiac trunk and the bifurcation is proportional to body size. The segmentation of the aorta was conducted by a third-year radiology resident. A second observer repeated 102 of the measurements.

**Fig. 2:**
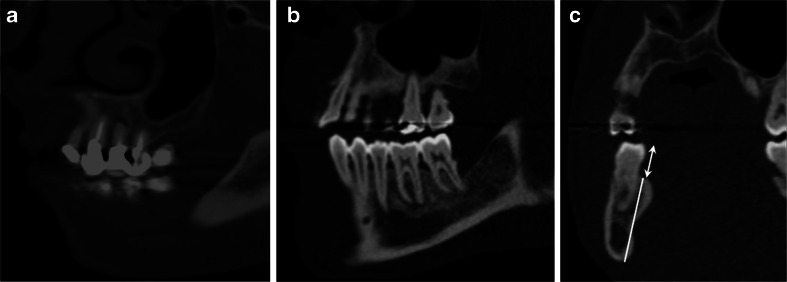
A typical CAP lesion of a tooth (14) in a semi-coronal reconstruction of a CT. The tooth shows an endodontic filling (**a**), a CAP lesion of a tooth (46) in a semi-sagittal reconstruction of a CT (**b**), and a semi-coronal reconstruction in the region 46 (**c**), showing the methods of measurement of the distance between the crown and the alveolar ridge (*double arrow*) and of measurement of the height of the bone (*white line*)

The jaws and teeth were evaluated without knowledge of the aortic atherosclerotic burden using a Picture Archiving and Communication System (IMPAX EE R20 VII, Agfa Health Care, Mortsel, Belgium) at a dedicated and approved high-resolution workstation by consensus of two investigators. A record was made of which tooth was located at which position, whether apical periodontitis was present or not, and whether the tooth had had endodontic treatment. To be assessed as CAP, there had to be a radiolucency associated with the root that was more than twice as wide as the adjacent periodontal gap on the crown side [23–25]. Figure 2a, b shows a typical CAP. If a tooth with more than one root had CAP on at least one root, it was considered to be affected by CAP. Since CAP can be a result of caries, restorative procedures, or trauma, the parameters that could be detected by CT were recorded qualitatively for each tooth (caries and/or fillings, yes or no).

Two different parameters for estimating horizontal bone loss were used to quantify marginal periodontitis. For this, the height of the alveolar ridge and the distance between crown and bone were measured in the regions of the interdental gap between teeth 11 and 21, 13 and 14, 16 and 17, 23 and 24, 26 and 27, 31 and 41, 33 and 34, 36 and 37, 43 and 44, and 46 and 47, as shown in Fig. 2c. The dental parameters were evaluated by consensus of a dentist and a board certified radiologist. Doubtful cases were decided by a third observer, a board certified radiologist. Another radiologist repeated the assessment of caries, restorations, root canal treatment, and CAP for 41 patients with 1,312 teeth. In order to assess reproducibility, kappa statistics were calculated for caries, restorations, root canal treatment, and CAP. Age and gender of the patients were also recorded.

Descriptive statistics were compiled using the Excel software (Microsoft, Seattle, WA, USA). The intra-observer and inter-observer variability for calcification of the aorta was determined based on the intraclass correlation coefficient (ICC) from Shrout and Fleiss [26]. Kappa statistics were calculated to determine this variability for categorical variables [27]. The unit of investigation was the individual. To illustrate this, first the atherosclerotic burdens were categorized in groups to show how many patients in these groups had CAP, root canal treatment, restoration, and caries. Qualitative properties were analyzed using the chi-squared test or Fisher’s exact test. Before further statistical analysis of the data, distribution analyses were made using the D’Agostino and Pearson tests. Since the data were not normally distributed, comparisons between two groups were made using the Mann–Whitney test while comparisons of several groups were made using the Kruskal–Wallis test. Spearman’s coefficient was used for correlation analyses. Because the distribution of aortic calcification data was not normal, but followed a complex unilateral function, a secondary parameter was formed in which all patients with no aortic calcification were assigned the value “0” and all patients with any amount of calcification were assigned the value “1.” Finally, with this parameter as a target variable, logistic regression models were fitted, first including all parameters measured and then in a stepwise forward selection procedure in order to identify other possible independent variables. The parameters for which the Wald test initially rejected the zero hypotheses were included in the final models. All analyses were made using GraphPad Prism software by GraphPad (San Diego, CA, USA) and SPSS 20 (IBM). A *p* < 0.05 was considered significant.

## Results

The 11,191 remaining teeth in the 531 patients were assessed. Table 1 shows the descriptive statistics of the analyzed parameters as well as the results of the tests for intra- and inter-observer variability. Reproducibility was very high for the assessment of the dental parameters and aortic atherosclerotic burden. There was no association between the diseases representing the indication for the examinations and the aortic atherosclerotic burden.

**Table 1:** Descriptive statistics of the analyzed parameters and the results of the tests for intra- and inter-observer variability

	Unit	Total	%	Subjects	%	Range
Teeth	Number	11,191	100	531	100	0–32
CAP	Number	1,269	11.3	389	73.25	0–15
Endo Tx	Number	827	7.38	291	54.8	0–13
ETx w CAP	Number	438	3.9	212	39.9	0–11
ETX w/o CAP	Number	389	3.5	209	39.4	0–9
CAP w/o ETX	Number	831	7.4	319	60.1	0–13
Fillings	Number	5,880	52.5	460	86.6	0–28
Caries	Number	4,031	36	459	86.4	0–30
Edentulous	Individual	(0)	(0)	50	9.4	(0–1)
Women	Individual			259	48.8	
Men	Individual			272	51.2	
Atherosclerosis	Individual			276	52.0	
		Mean	SD		Range	
Age	Years	50.0	15.7		8.1	89.9
Height of the alveolar ridge	mm	22.9	3.8		7	32.7
Distance between crown and bone	mm	9.3	1.9		0	16.5
Atherosclerosis	ml	0.28	0.83		0	8.52
Test of reliability	Unit	Rater	Number of cases	Kappa	SE	Degree of agreement
Cohen’s kappa	CAP (yes/no)	2	1,312 (teeth)	0.942	0.017	“Very good”
Cohen’s kappa	Endodontic treatment (yes/no)	2	1,312 (teeth)	1	0	“Perfect”
Cohen’s kappa	Caries (yes/no)	2	1,312 (teeth)	0.977	0.006	“Very good”
Cohen’s kappa	Fillings (yes/no)	2	1,312 (teeth)	0.998	0.002	“Very good”
Cohen’s kappa	Atherosclerosis (yes/no)	2	80 (patients)	1	0	“Perfect”
ICC	Atherosclerosis (ml)	2	102	0.9753		“Very good”

**Fig. 3:**
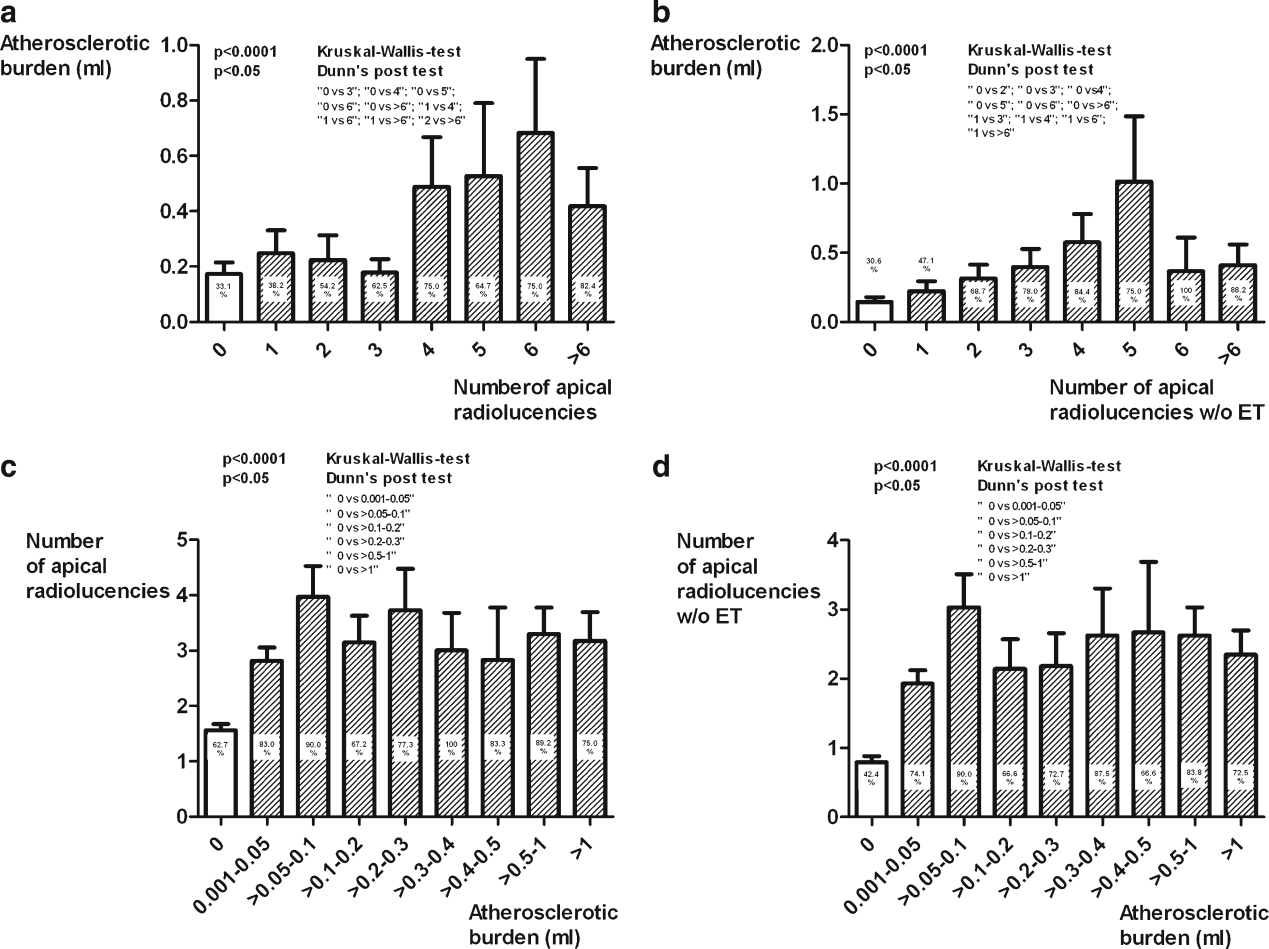
CAP lesions on the abscissa, versus the aortic atherosclerotic burden on the axis of ordinates (*bars*), and the percentage of patients with quantifiable atherosclerotic lesions denoted as numbers (**a**); CAP lesions without endodontic therapy on the abscissa, versus the aortic atherosclerotic burden on the axis of ordinates (*bars*), and the percentage of patients with quantifiable atherosclerotic lesions denoted as numbers (**b**); the aortic atherosclerotic burden, grouped on the *x*-axis, versus the number of apical radiolucencies on the axis of ordinates (*bars*), and the percentage of patients with apical radiolucencies denoted as numbers (**c**); the aortic atherosclerotic burden, grouped on the *x*-axis, versus the number of apical radiolucencies without endodontic therapy on the axis of ordinates (*bars*), and the percentage of patients with apical radiolucencies denoted as numbers (**d**)

Comparisons between two groups, and correlations between the different parameters, and the aortic atherosclerotic burden are shown in Table 2. The patients with at least one CAP lesion had a considerably higher aortic atherosclerotic burden than patients without CAP, and the patients with at least one root canal treatment had a considerably lower aortic atherosclerotic burden than patients with no root treatment. Women had a lower aortic atherosclerotic burden than men. The surrogate parameter for marginal periodontitis, the distance between crown height and bone, correlated significantly with aortic atherosclerotic burden. The more CAP lesions were present in a patient, the greater the probability of detecting quantifiable atherosclerotic lesions and the greater the aortic atherosclerotic burden were (Fig. 3a, b). Figure 3c, d shows the number of CAP lesions related to the extent of the aortic atherosclerotic burden.

**Table 2:** Comparisons between two groups, and correlations between the different parameters, and the aortic atherosclerotic burden

	Individuals	Individuals with ATS	*p*	Test	Abdominal atherosclerotic burden (ml)	SD	*p*	Test
Women	259	118	0.0055	FET	0.23	0.75	0.0060	MW
Men	272	157			0.33	0.9		
At least 1 CAP	389	228	<0.0001	FET	0.32	0.92	<0.0001	MW
No CAP	142	47			0.17	0.51		
At least 1 root canal treatment	291	147	0.5419 (ns)	FET	0.15	0.52	<0.0001	MW
No root canal treatment	240	128			0.44	1.01		
At least 1 apical radiolucency with endodontic treatment	212	108	0.7904 (ns)	FET	0.18	0.6	0.2568 (ns)	MW
No apical radiolucency with endodontic treatment	319	167			0.34	0.95		
At least 1 apical radiolucency w/o endodontic treatment	319	210	<0.0001	FET	0.37	1	<0.0001	MW
No apical radiolucencies w/o endodontic treatment	212	65			0.14	0.48		
At least 1 root canal treatment w/o apical radiolucency	210	104	0.4246 (ns)	FET	0.15	0.43	0.0297	MW
No root canal treatment w/o apical radiolucency	321	171			0.36	0.99		
0–5 fillings or crowns	143	90	0.0023	FET	0.54	1.05	<0.0001	MW
>5 fillings or crowns	388	185			0.18	0.72		
0–5 carious lesions	277	145	0.7949 (ns)	FET	0.33	0.90	0.3356 (ns)	MW
>5 carious lesions	254	130			0.22	0.76		
0–5 carious lesions (without edentulous individuals)	227	99	0.1007 (ns)	FET	0.18	0.70	0.0735 (ns)	MW
>5 carious lesions (without edentulous individuals)	154	120			0.22	0.76		
Edentulous patients	50	46	<0.0001	FET	0.97	1.32	<0.0001	MW
Not edentulous patients	481	229			0.21	0.73		
		Spearman’s *ρ*	95 % Confidence interval (lower margin)		95 % Confidence interval (upper margin)		Significance	
Age	Years	0.7471	0.7057		0.7835		<0.0001	
Teeth	Number	−0.5678	−0.6181		−0.4977		<0.0001	
Apical radiolucencies	Number	0.3230	0.2423		0.3993		<0.0001	
Endodontic therapies	Number	−0.07370	−0.1603		0.01400		0.0898 (ns)	
Apical radiolucencies with endodontic therapy	Number	−0.02777	−0.1151		0.05999		0.5232 (ns)	
Apical radiolucencies w/o endodontic therapy	Number	0.4132	0.3378		0.4833		<0.0001	
Endodontic therapies w/o apical radiolucencies	Number	−0.08957	−0.1758		−0.01966		0.0391	
Restorations (fillings and/or crowns) per tooth	Number	−0.2359	−0.3170		−0.1514		<0.0001	
Caries	Number	−0.06734	−0.1540		0.02040		0.1212 (ns)	
Caries per tooth	Number	0.1106	0.02305		0.1965		0.0109	
Restorations (fillings and/or crowns) per tooth	Number	0.02360	−0.06431		0.1112		0.5881 (ns)	
Endodontic therapies per tooth	Number	−0.01431	−0.1020		0.07357		0.7427 (ns)	
Height of the alveolar ridge	mm	0.07091	−0.01933		0.1600		0.1126 (ns)	
Distance from the crown to the bone	mm	0.4080	0.3322		0.4785		<0.0001	

*MW* Mann–Whitney, *FET* Fisher’s exact test

Table 3 shows the aortic atherosclerotic burden as a function of various combinations of CAP lesions and endodontic treatment. Patients with CAP lesions without endodontic treatment had a greater aortic atherosclerotic burden than patients with CAP lesions with endodontic treatment.

**Table 3:** Aortic atherosclerotic burden as a function of various combinations of CAP lesions and endodontic treatment

Apical radiolucencies	Root canal treatments	Apical radiolucencies w/o root canal treatment	Number of patients	Number of patients with atherosclerosis	Patients with atherosclerosis (%)	Atherosclerotic burden	SD
0	0	0	112	36	32.1	0.2147	0.5688
0	1 or more	0	30	11	36.6	0.0163	1.0457
1	0	1	45	22	48.9	0.5130	1.2070
1	1	0 or 1	27	7	25.9	0.0388	0.1336
1	2 or more	0 or 1	30	10	33.3	0.0402	0.1379
2	0	2	26	21	80.8	0.5752	1.4017
2	1 or 2	0, 1, or 2	35	16	45.7	0.0687	0.1930
2	3 or more	0, 1, or 2	22	8	36.4	0.0542	0.1441
3	0	3	18	16	88.9	0.5603	0.6926
3	1 or 2	1, 2, or 3	25	14	56	0.0655	0.1857
3	3	0, 1, or 2	14	7	50	0.0404	0.0779
3	4 or more	0, 1, or 2	15	8	53.3	0.0335	0.0789
4	0	4	14	13	92.9	0.9623	1.6128
4	1–4	0–4	21	15	71.4	0.1248	0.2138
4	5 or more	0–1	5	2	40	0.6872	1.5276
5	0	5	12	9	75	1.0164	2.4437
5	1–5	2–5	16	9	56.3	0.1174	0.2511
5	6 or more	0–2	6	4	66.7	0.639	1.0816
6	0	6	4	4	100	0.7657	1.1968
6	1–6	0–6	13	11	84.6	0.8148	1.3309
6	7 or more	1–2	3	0	0	0	0
7	0	7	7	6	85.7	0.4957	0.7113
7	1–7	3–7	11	9	81.8	0.6205	1.3143
≥8	0–13	1–13	20	17	85	0.2786	0.5540

In all logistic regression models, the age of the patients was the factor with by far the greatest positive effect on the aortic atherosclerotic burden. The maximum likelihood estimation for age, at theta = 90.8, was greater by a factor of 4 than the factor with the second greatest magnitude, CAP without endodontic treatment, at theta = 23.9. Male gender was another independent factor that was positively correlated to the aortic atherosclerotic burden in the logistic regression models. The predominance of the effect of the factor “age” and strong collinearity with this factor had an effect on the factors distance between the highest point of the crown and the alveolar ridge as a surrogate for periodontitis. In models that were not corrected for the factor age, this factor had a statistically significant effect on the atherosclerotic burden (Table 4). Neither root canal fillings in teeth without apical radiolucencies nor apical radiolucencies associated with teeth with root canal fillings were found to be important factors for the aortic atherosclerotic burden. The parameters included explained 63.6 % of the variability of the aortic atherosclerotic burden. CAP without endodontic treatment on its own explained 11.4 % of the variability.

**Table 4:** Logistic regression model with the target variable “existence of aortic calcifications” using standard criteria for inclusion and exclusion (model 1) and logistic regression model with the target variable “existence of aortic calcifications” disregarding the factor “age” (model 2)

	Wald	Significance	Regression coefficient (*β*)	Exp (*β*)	Lower 95 % CI	Upper 95 % CI
Model 1						
Age	90.785	<0.001	0.096	1.101	1.079	1.123
Number of apical radiolucencies w/o endodontic therapy	23.964	<0.001	0.385	1.470	1.260	1.715
Gender (male)	7.404	0.002	0.631	1.879	1.193	2.960
Caries per tooth	6.827	0.009	1.073	2.924	1.308	6.540
Number of restorations (fillings and/or crowns)	4.725	0.030	−0.37	0.964	0.933	0.996
Constant	79.444	<0.001	−6.092	0.002		
Model 2						
Number of teeth	55.967	<0.001	−0.095	0.909	0.887	0.932
Caries per tooth	9.017	0.003	1.076	2.933	1.453	5.919
Number of restorations	10.992	0.001	−0.048	0.953	0.927	0.981
Number of apical radiolucencies w/o endodontic therapy	39.953	<0.001	0.474	1.606	1.387	1.860
Distance from the crown to the bone	17.836	<0.001	0.259	1.295	1.148	1.459
Gender	4.79	0.029	0.439	1.551	1.047	2.296
Constant	24.509	<0.001	0.689	3.413	–	–

The surrogate parameter for marginal periodontitis appears within the models

## Discussion

The results of this study clearly demonstrate a positive correlation, independent of the effect of marginal periodontitis and caries, between CAP and the aortic atherosclerotic burden. Only CAP without endodontic treatment was an important factor for the aortic atherosclerotic burden, not apical radiolucencies on teeth with endodontic treatment. The factor CAP without endodontic treatment is more significant than gender, marginal periodontitis, and caries and about one fourth as significant as age.

Since the aim of endodontic treatment of pulpitis, the main cause of CAP, is to control local infection and inflammation in order to preserve the tooth, the inverse correlation with atherosclerosis and the significance of CAP without endodontic treatment are very plausible in view of the hypothesis that inflammation is associated with atherosclerosis [28].

However, the study has some weaknesses and limitations that must be considered. Given the retrospective cross-sectional study design, the question of causality must remain unanswered and can be clarified only by further research. The study may be subject to an uncontrollable bias that can occur in retrospective studies, and the fact that the study design is retrospective limits its impact. The method used to quantify the aortic atherosclerotic burden [21, 22] underestimates the actual atherosclerotic burden because it detects only mature plaques, not so-called soft plaques. Most of the variability of the aortic atherosclerotic burden was explained by the parameters examined; the remaining 36.4 % variability must still be considered large. In this context, it must also be taken into consideration that this study was a retrospective proof-of-principle study. In our opinion, the prospective study that is ultimately needed would not have been approved without this retrospective observational study because the effort and potential damage from examinations conducted merely for the purpose of the study could not have been justified based only on a theoretical benefit for patients. Only a prospective design will make it possible to prove the existence of the effect and estimate its magnitude, taking all other risk factors into consideration.

In addition to marginal periodontitis, caries, and CAP, this prospective observational study must include all inflammatory diseases of the oral cavity that can be quantified and evaluated using objective criteria. On the one hand, this involves the clinical evaluation of pocket probe depth, caries, and restorations, but on the other hand also includes X-rays to assess CAP and the atherosclerotic burden of the patients. All potential cardiovascular risk factors would have to be considered. Such a study is conceivable only with a cross-sectional design because in the event caries or periodontitis are detected, a treatment recommendation would have to be given.

The strength of this study is that the method of calcium scoring used [21, 22] objectively quantifies the hard plaques of the aorta. Its objectivity sets it apart from all other methods of quantification. The study included a large number of consecutive patients who were not being treated for either a dental or a cardiovascular problem, but for conditions that occur independently of diseases of the teeth and jaws or the cardiovascular system.

The designs of the studies that yielded earlier evidence of possible correlations between periodontitis or apical periodontitis and atherosclerosis should be viewed more closely. The 1989 study by Mattila et al. [6] was a prospective case–control study that was carried out to examine the observation that “chronic dental infections” were “common among patients with acute myocardial infarction.” The cross-sectional study by Beck et al. [8] conducted in the USA, a prospective observational study of 6,017 patients at one time point, and the prospective longitudinal study over an observational period of 16 years by Söder et al. [9] conducted in Sweden both resulted in a correlation between marginal periodontitis and manifestations of atherosclerosis in the carotid artery. The information on the progression of carotid atherosclerosis depending on the index of decayed, missing, and filled teeth and on the Silness–Löe Index also stems from a prospective, longitudinal, observational study [10].

By contrast, the first study that examined a possible correlation between periapical disease and coronary heart disease was a retrospective, cross-sectional study. The later studies were also either retrospective [15, 16] or retrospectively used data sets that were originally gathered prospectively [15, 16]. One study had a mixed design with retrospective and prospective elements [17], and only one study had a prospective design [19]. The association found between apical periodontitis and coronary heart disease in this latter case–control study may, however, be attributed to that fact that cardiovascular diseases were excluded in the control group, giving the study an uncontrollable source of error. While the correlation between marginal periodontitis and atherosclerosis is well documented [8–10] and is also reflected in the data here based on the proven significance of horizontal bone loss, the same does not apply to caries. The reason for this may be that in early studies, caries and periapical lesions were included along with marginal periodontitis and pericoronitis [6, 29], so that it was ultimately impossible to distinguish which of the diseases of the teeth and their supporting structures were possibly associated with the risk and which were not. CAP as a consequence of severe penetrating caries [14] is a possible cause of tooth loss [30, 31], but both partial and total edentulism seem to be associated with coronary heart disease and stroke themselves [32–35]. This study confirmed the pronounced association between edentulism and atherosclerosis. The more teeth were missing, the greater was the chance of having abdominal aortic calcification. However, this association had no effect on the logistic regression models accounting for age. This leads to the conclusion that it is probably not lack of teeth per se that is associated with a more pronounced atherosclerotic burden but that edentulism must be considered to be merely an epiphenomenon of CAP that can be readily assessed clinically.

The significance of the factor “age” in the logistic regression analyses merits a closer look. In univariate analyses, age correlated with almost all other factors, in particular with edentulism, but also with the number of teeth affected by CAP, root canal treatment, and the aortic atherosclerotic burden. Omitting age led to entering other factors—marginal periodontitis, and number of teeth—into the models. Their introduction into the models after the omission of age can be explained by existing collinearities. Another explanation for the phenomenon is the fact that the magnitude of the significance of age is several times greater than that of the other factors. This difference could result in a method-related underestimation of the significance of “subordinate” factors. It should also be taken into consideration that initial changes such as stage 2 gingivitis or gingival recession [36] are difficult to detect in the CT. The method applied in our study measured only the irreversible changes in stages 3 and 4 periodontitis that are associated with horizontal bone loss. Marginal periodontitis was thus certainly underestimated.

The analysis of the subgroups of patients with the various possible combinations of lesions is quite interesting. Patients with one or several teeth affected by CAP without endodontic treatment had a higher atherosclerotic burden than patients with one or more teeth affected by CAP who had had endodontic treatment on the same tooth or teeth. The atherosclerotic burden was the lowest in patients with one or more endodontic treatments with no CAP. Irrespective of the apical status, patients with root canal fillings thus had a lower atherosclerotic burden. Moreover, the more CAP lesions were present, the more likely the patient was to manifest aortic calcifications. Of course, ultimately no causalities can be proven on the basis of a retrospective study such as this. However, considering that the aim of endodontic treatment is to reduce pulpal infection [12–14], the possibility must be kept in mind that pulpal infection and inflammation, for example associated with severe caries and the resulting CAP with accompanying local osteitis, is a factor promoting atherosclerosis that could be effectively eliminated by endodontic treatment. However, due to the retrospective design of this study, this hypothesis must be verified by further investigations. New in any event is the unbiased recording of the association between CAP without endodontic treatment and atherosclerotic burden, the strength of the association between the two, and the missing association between CAP with endodontic treatment and atherosclerosis, i.e., a separation of a history of disease from the disease itself.

The large number of subgroups of patients with various combinations of CAP on one or more teeth and root fillings on one or more roots of the same tooth or different teeth makes the comparison of individual groups extremely difficult. Some patients had teeth with and without CAP and with and without various numbers of root canal fillings in the same jaw. The atherosclerotic burden fluctuated among these groups, as can be seen in Table 3, so that comparisons of all subgroups of patients were unfeasible. Only the aggregation of the numerous subgroups and the variance and regression analyses used here that included the various factors that were present to a different degree in each patient appeared to be adequate for analyzing the data.

Endodontic treatment has previously been considered to be an expression and consequence of poor oral health in patients with myocardial infarction [37]. With this in mind, endodontic treatment was used as a surrogate parameter for the presence of endodontic disease in the retrospective analysis of the data from the Atherosclerosis Risk in Communities study to investigate the correlation with coronary heart disease, although endodontic therapy represents treatment of endodontic disease more than being a manifestation of disease itself [16]. In the Health Professionals Follow-Up Study, endodontic treatment was used in conjunction with CAP as an indication of the presence of pulpal infection, so the effect of CAP on the probability of coronary heart disease, with an odds ratio of 1.21, was probably underestimated in this study [17]. The suspicion that CAP treatment already carried out due to pulpal inflammation may have hindered the detection of the correlation with coronary heart disease was first expressed after the retrospective analysis of the data from the VA Dental Longitudinal Study and the Normative Aging Study [15] and was confirmed in our study. Furthermore, the observation of the neutralization of the association between apical radiolucencies and atherosclerosis through endodontic treatments allows the hypothesis to be made here that endodontic treatment whose aim is to eliminate infection should not be viewed merely as a tooth-preserving measure, but may also provide protection against atherosclerosis. This entirely new approach would be greatly significant given the extent of the consequences of atherosclerosis, as ischemic heart disease and cerebral vascular disease are the greatest leading causes of death in the Western world [38]. The hypothesis that endodontic treatment could have a protective effect with respect to atherosclerosis must be tested in other studies. However, the findings described here should be grounds for providing dental or periodontal examinations to patients with a high atherosclerotic burden who may even have already suffered a cardiovascular event. The finding that caries correlates positively and fillings correlate inversely with the aortic atherosclerotic burden confirms the results of a recently published study [20]. The finding that caries has an effect in the regression models independently of CAP could be explained by the fact that deep carious lesions are the main cause of pulpal infection and inflammation. In some cases, pulpal infection may be present before CAP has developed. Nevertheless, the infection and inflammation in the pulp is already in contact with the bloodstream. Analogous to endodontic treatment for CAP, filling carious lesions reduces infection and inflammation and protects against the inflammatory process.

The percentage of teeth affected by apical radiolucencies was 11.4 %, somewhat above average when compared with the data from other studies, which reported between 2 and 13.6 %. By contrast, the number of teeth that had undergone root canal treatment (7.4 %) was about average in comparison with literature, where 1.3 to 22.8 % was reported [39]. The good comparability of the results with those from literature allows the conclusion to be made that the reliability of the data is good, although the maximum resolution of the modern multidetector CT scanner used here does not quite reach that of cone beam computed tomography [25]. However, the local resolution was still far better than that for which superiority compared with intraoral X-rays has been proven [40].

The 21.0 ± 9.8 teeth per patient found were within the average found in other studies [40], and the distribution of CAP and endodontic treatment to molars, premolars, and anterior teeth was consistent with data from literature [41]. The greater number of teeth in the lower jaw compared with the upper jaw, the lower number of CAP lesions [39], and the lower number of teeth with root treatment in the lower jaw were also consistent with data from literature [42]. For this study, this can considered to be an indicator of the reliability of the data, but discussing these findings would digress from the topic.

It is known that a large percentage of root-filled teeth are likely to be associated with CAP. CAP is the visible X-ray manifestation of inflammation in a process whose cause is generally pulpal infection, usually as a result of deep caries. The process spreads to the apical region and ultimately leads to local osteitis and osteomyelitis. The cancellous bone is absorbed and replaced by granulation tissue and accompanying infection and inflammation products. The defect in the cancellous bone is manifested as radiolucency in the X-ray. Ideally, after endodontic treatment, the infection is eliminated and the inflammation is no longer present. If there is no regeneration of the apical defect in the cancellous bone, the defect remains but is no longer a focus of inflammation. This could explain why the aortic atherosclerotic burden is just as high in patients with root fillings and apical radiolucencies on the same tooth as in patients with root fillings without apical radiolucencies.

In summary, this study shows that CAP without endodontic treatment—not however CAP with endodontic treatment—correlates with the aortic atherosclerotic burden. There is a clear inverse correlation between endodontic treatment and the aortic atherosclerotic burden. In view of the importance of coronary heart disease and cerebrovascular disease that are consequences of atherosclerosis, prospective studies to evaluate the causality are extremely important. If causality is confirmed, endodontic treatment could achieve an entirely new status in the secondary prevention of cardiovascular events.
